# Hidradenitis suppurativa, from basic science to surgery and a new era of tailored targeted therapy: An expert opinion paper

**DOI:** 10.1007/s00403-025-04016-1

**Published:** 2025-02-28

**Authors:** Angelo Valerio Marzano, Michele Bartoletti, Vincenzo Bettoli, Luca Bianchi, Andrea Chiricozzi, Mario Clerici, Paolo Dapavo, Valentina Dini, Caterina Foti, Cristina Magnoni, Matteo Megna, Giuseppe Micali, Elisa Molinelli, Francesca Prignano

**Affiliations:** 1 Dermatology Unit, Fondazione IRCCS Ca’ Granda Ospedale Maggiore Policlinico, Milan, Italy; 2 Department of Pathophysiology and Transplantation, Università degli Studi di Milano, Milan, Italy; 3 Department of Biomedical Sciences, Humanitas University, Milan, Italy; 4 Infectious Disease Unit, IRCCS Humanitas Research Hospital, Rozzano, Italy; 5 O.U. Of Dermatology, Department of Medical Sciences, University of Ferrara, Ferrara, Italy; 6 Dermatology, Fondazione Policlinico Tor Vergata, Department of Systems Medicine, Tor Vergata University, Rome, Italy; 7 Dermatologia, Dipartimento Universitario di Medicina e Chirurgia Traslazionale, Università Cattolica del Sacro Cuore, Rome, Italy; 8 U.O.C. Dermatologia, Dipartimento di Scienze Mediche e Chirurgiche, Fondazione Policlinico Universitario A. Gemelli IRCCS, Rome, Italy; 9 Università Degli Studi Di Milano, Milan, Italy; 10 Don C Gnocchi Foundation IRCCS, Milan, Italy; 11 Azienda Ospedaliero Universitaria Città della Salute e della Scienza di Torino, Turin, Italy; 12 Università di Pisa, Pisa, Italy; 13 Università degli Studi di Bari, Bari, Italy; 14 Surgical, Medical and Dental Department of Morphological Sciences Related to Transplant, Oncology and Regenerative Medicine, University of Modena and Reggio Emilia, Modena, Italy; 15 Section of Dermatology, Department of Clinical Medicine and Surgery, University of Naples Federico II, Naples, Italy; 16 Dermatology Clinic, University of Catania, Catania, Italy; 17 Department of Clinical and Molecular Science, Dermatologic Clinic, Azienda Ospedaliero Universitaria delle Marche, Ancona, Italy; 18 Department of Health Sciences, Dermatology Section, Università degli Studi di Firenze, Florence, Italy

**Keywords:** Hidradenitis suppurativa, Interleukin 17, Secukinumab, Targeted therapy, Unmet needs

## Abstract

Hidradenitis suppurativa (HS) is a chronic inflammatory disease characterised by an aberrant activation of innate immunity and increased production of pro-inflammatory mediators such as interleukin 17 (IL-17). IL-17 has been shown to play a key role in the pathogenesis of HS and evidence highlights the potential of IL-17-targeted therapies. The fully human IgG/κ monoclonal antibody secukinumab, which specifically targets IL-17A and inhibits interaction with its receptor, has recently been approved for the treatment of moderate-to-severe HS. Secukinumab offers patients an efficacious and well-tolerated treatment option in terms of sustained response by rapidly improving signs and symptoms, and preventing disease progression in the absence of loss of response. Being a challenging disease, HS is associated with a delay to diagnosis of 3–10 years and, consequently, late implementation of appropriate treatment, leading to disease progression. Misdiagnosis due to flawed understanding and lack of awareness among medical providers and patients is considered an important factor contributing to the delayed diagnosis. Thus, serious efforts must be made on a large scale to urgently reduce the delay in HS diagnosis and reduce the disease burden in patients, including raising awareness, implementation of education programmes at medical and specialisation schools, as well as continuous education of healthcare providers at different levels for the early detection of HS and initiation of appropriate treatment. Here, we present the main critical unmet needs in the diagnosis and treatment of patients affected by HS, address how disease awareness and comprehensive multidisciplinary management (offering both medical and surgical care) can benefit patients, and suggest therapeutic options, based on clinical characterisation and early identification and intervention (window of opportunity), to be adopted for a timely and better management of disease progression and to fill current gaps.

## Introduction

Hidradenitis suppurativa (HS) is a painful, non-contagious chronic inflammatory skin disease [1] affecting primarily apocrine gland-rich areas of the body, presenting painful nodules and abscesses that lead to the formation of sinus and fistula tracts, and scarring upon rupture [2, 3]. Affected body parts are the terminal hair follicle units of the axilla, inframammary folds, groin and buttocks [4]. Due to its chronicity and the deep painful lesions, HS has a substantial negative effect on patients’ quality of life [5]. The prevalence of HS is ~ 1% and the disease typically begins during or after puberty [6].

A plethora of factors are believed to cause HS: genetics, the cutaneous microbiome, immunologic factors and hormonal predisposition, as well as environmental factors such as obesity, smoking and skin occlusion [3, 7–10], translating into phenotypic disease heterogeneity and a variable response to therapy [11].

HS belongs to the heterogenous group of neutrophilic dermatoses (inflammatory skin disorders), which are characterised by sterile neutrophilic infiltrates in the skin [12, 13]. In patients with HS, neutrophil extracellular trap (NET) formation is enhanced in circulating neutrophils, which undergo spontaneous NETosis [14]. Moreover, NETotic neutrophils have been found in HS lesions, particularly in the lesional tunnel [15], as well as a high number of polymorphonuclear cells [16]; importantly, a positive correlation between the amount of NETs within skin lesions and HS disease severity has been reported [17].

HS is characterised by aberrant activation of innate immunity [18] and a large inflow of pro-inflammatory mediators (i.e. interferon [IFN]-γ, tumour necrosis factor [TNF]-α, interleukin [IL]−6, −8, −17 and −12/23) [19–21]. Three main events occur in HS pathogenesis, namely (i) follicular occlusion, (ii) rupture of the dilated follicle and (iii) chronic inflammation with sinus tract formation, each of which is characterised by a cascade of events [19]. Follicular occlusion is defined by hyperkeratosis and hyperplasia of the follicular epithelium, causing the formation of a keratin plug due to the accumulation of cellular debris. Rupture of the dilated follicle follows, with dispersion of keratin fibres, bacteria and pathogen- and damage-associated molecular patterns (PAMPs/DAMPs) into the dermis, triggering an acute and severe immune response that induces a large inflow of immune cells and the release of cytokines including IL-1β and TNF-α [12]. This can lead to the formation of nodules, abscesses or fistulas. Subsequently, chemokines, such as CXCL8, CXCL11, CCL2 and CCL20 in keratinocytes, and CXCL1 and CXCL6 in fibroblasts are produced [12], recruiting in turn more inflammatory cells. Importantly, at this stage, activated dendritic cells produce IL-12, inducing T helper (Th) 1 polarisation, and IL-23, which maintains the Th17 phenotype. Lastly, chronic inflammation is characterised by the formation of epithelialised tunnels, sinus tracts and keloid scars, further triggering inflammation and the production of IFN-γ (which recruits more inflammatory cells) and TNF-α (which supports Th17 polarisation), among others. Th17 cells are characterised by the production of IL-17 [22] that, in turn, stimulates neutrophil- and macrophage-induced production of IL-1β, IL-6, TNF-α and matrix metalloproteinases, and, consequently, the formation of fibrosis and sinus tracts [19].

Several staging systems have been developed for the assessment of HS: the Hurley staging system [23]; Hidradenitis Suppurativa Clinical Response (HiSCR) [24]; Hidradenitis Suppurativa Physician Global Assessment [25, 26]; Hidradenitis Suppurativa Severity Index [26, 27]; Modified Sartorius Score [28, 29]; and International Hidradenitis Suppurativa Severity Score System (IHS4) [30] and its dichotomous version IHS4-55 [31, 32]. Each of these systems serves a different purpose, such as assessment of disease severity (e.g. mild, moderate and severe) or efficacy of intervention [19, 33].

Despite recent advances, and the fact that HS was first described in the mid-1800s, the pathogenesis is not fully understood, and the main challenges remain disease management and treatment [2]. In fact, patients experience significant physical and emotional burdens, with HS negatively impacting their lives and well-being [3].

There are a number of limitations to the treatment of HS, namely diagnostic delay (ranging from 3 to 10 years); misdiagnosis, associated with diagnostic delay due to flawed understanding and lack of disease awareness among non-dermatologic providers; the progressive nature of HS [34]; and, importantly, management guidelines*,* which generally present agreement only on first-line treatments due to disease heterogeneity [35, 36]. The recently published German S2k guidelines on HS therapy address some of these issues [37], and European guidelines are being developed.

Currently, HS treatment therapy includes topical, systemic, surgical and combined treatment, which can help manage the disease [19, 37]. Conventional therapies (oral antibiotics and topical treatments) are used to treat mild and moderate HS, and as adjuvant treatment in moderate-to-severe HS, although some patients show resistance to such treatment [33, 38]. More recently, biological therapy has become a promising option in the treatment of HS, with adalimumab (a TNF-α inhibitor), secukinumab (an IL-17A inhibitor) and bimekizumab (an IL-17A and −17F inhibitor) now approved for the treatment of HS. Several other targeted therapies (such as IL-1, IL-6 and IL-23 inhibitors, and Janus kinase [JAK] inhibitors) may be used off-label and have shown variable clinical response [33, 39]. Despite a significant improvement in clinical outcomes upon treatment with adalimumab [40], the first biological agent approved for the treatment of moderate-to-severe HS, not all patients achieve a primary response and some develop a secondary loss of response; thus, its long-term effectiveness is highly variable [19]. Nonetheless, biological agents are used in patients with severe HS who show a suboptimal response to these agents in order reduce the area required for surgical resection [33, 41]. Indeed, a better outcome has been observed in patients with moderate-to-severe HS undergoing a combination of adalimumab treatment and surgery compared with adalimumab alone [42, 43].

In 2023, both the European Medicines Agency (EMA) and the United States (US) Food and Drug Administration (FDA) approved secukinumab for the treatment of moderate-to-severe HS in adults [44], based on two major phase III, randomised, controlled clinical trials that showed promising results with a favourable safety profile and sustained response for up to 52 weeks [45]. The IL-17A and −17F inhibitor bimekizumab is approved for HS in Europe and is undergoing regulatory review for this indication in the US.

The aim of this paper is to describe the expert authors’ opinions of the current management of HS, as well as the pathogenic role of IL-17 in HS and emerging targeted therapies, with a focus on secukinumab. We also aim to define the components of a comprehensive multidisciplinary team (MDT), which is indispensable for the optimal management of HS, both in terms of early diagnosis and early implementation of personalised therapy.

## Current and future treatments in the management of HS

### Antibiotic therapy

Antibiotic therapy has been shown to be an effective treatment option for patients with HS and, in fact, antibiotics are recognised as first-line treatments [38, 46]. In the context of HS, antibiotics are administered primarily for their anti-inflammatory effects, rather than their antimicrobial properties.

The recently published German S2k guidelines are aimed at aiding the selection and implementation of suitable/sufficient therapy for HS [47]. Specifically, these guidelines recommend doxycycline as first-line treatment, while clindamycin treatment, either as monotherapy or in combination with rifampicin, is listed as ‘should be recommended*’* or second-line treatment [47].

Based on their clinical experience, the experts’ provided their opinions on the initial approach to the use of antibiotics to treat HS, including the duration of treatment, empirical versus targeted therapy and on-going therapy (Table 1).

**Table 1:** Expert opinion on the use of antibiotics in the management of hidradenitis suppurativa

**Initial approach**
The initial approach in the treatment of HS must be antibiotic therapy as first-line treatment, i.e. tetracycline (doxycycline) or clindamycin [47]
Compared with the combination of rifampicin and clindamycin, tetracyclines seem to have a similar efficacy but are associated with fewer adverse effects and pharmacological interactions [38, 79]
Rifampicin, specifically, is associated with significant enzymatic drug interactions, which can interfere with plasma levels of concomitant clindamycin [80], so careful attention should be paid to pharmacological interactions when rifampicin is used [38, 81, 82]
It may be advisable to try clindamycin monotherapy before prescribing the combination of clindamycin + rifampicin since there appears to be little difference in outcomes between the two approaches [83]
Duration of treatment
The duration of antibiotic treatment should be 8–12 weeks
Currently, however, there is an urgent need to identify, at an early stage, patients who do not respond to antibiotic treatment, in order to offer these patients second-line treatments
Thus, moderate-to-severe forms of HS that are non-responsive to 4–6 weeks of antibiotic therapy and/or relapse should be treated with biological therapies (anti-TNF-α or anti-IL-17)
For example, a patient who is being assessed at a dermatology centre for the first time but who has previously received several lines of antibiotics would not need a repeat course of antibiotics, especially if they have a long disease history
Empirical versus targeted therapy
Empirical antibiotic treatment is undoubtedly the first choice in HS, but targeted therapy may be indicated in some selected cases, when the specific acute clinical features suggest a possible superinfection by pathogenic bacteria
However, microbiological sampling can be problematic because it is difficult to obtain representative and ideal sampling of the main pathogen, using both biopsies and cultures, due to a highly contaminated context, both after surgery or independently of surgery
Targeted antibiotic therapy should take account of antimicrobial resistance in sample isolates; rates of tetracycline resistance can be high in pathogens isolated from HS lesions, as can resistance to other antibiotics [81, 84]
Ongoing therapy
HS pathology has a variable clinical picture: there are phases that are fairly calm and phases with flare-ups, as well as phenotypes characterised either by predominant inflammation or follicular occlusion
Such sudden and extreme variabilities require adaptation of antibiotic treatment based on the clinical phase

*HS* hidradenitis suppurativa, *IL* interleukin, *TNF-α* tumour necrosis factor alpha

### Biological therapy

The approved biological therapy options for HS in Europe are adalimumab, secukinumab and bimekizumab (Table 2) [48–50]. Secukinumab and bimekizumab are approved for the treatment of adult patients with moderate-to-severe active HS who have had an inadequate response to conventional treatment [48, 50]. Adalimumab is approved for adults and adolescents aged ≥ 12 years with moderate-to-severe active HS [49]. The 2024 S2k German guidelines for HS, which predated the European approval of bimekizumab for HS, recommend adalimumab or secukinumab for patients with moderate-to-severe HS, with the option of off-label use of intravenous infliximab or bimekizumab [47]. The efficacy of biological treatment should be reviewed after 12 weeks (adalimumab) or 16 weeks (bimekizumab and secukinumab) and treatment switched or discontinued if suboptimally effective.

**Table 2:** Biological therapy approved in Europe for the treatment of HS

Biological agent	Mechanism of action	Approved indication	Route of administration	Dose	Review for response
Adalimumab [49]	TNF-α inhibitor	Active moderate-to-severe HS in adults and adolescents aged ≥ 12 years	SC	Adults: 160 mg on day 1, 180 mg on day 15, then 40 or 80 mg every 2 weeks from day 29 Adolescents: 80 mg on day 1 then 40 mg every other week starting at week Option to increase dose to 80 mg every other week for inadequate response	12 weeks
Secukinumab [48]	IL-17A inhibitor	Active moderate-to-severe HS in adults with an inadequate response to conventional systemic therapy	SC	300 mg/week at weeks 0, 1, 2, 3 and 4 then 300 mg/month. Option to increase dose to 300 mg every 2 weeks for inadequate response	NS, but clinical trials used 16 weeks
Bimekizumab [50]	IL-17A, IL-17F and IL-17AF inhibitor	Active moderate-to-severe HS in adults with an inadequate response to conventional systemic therapy	SC	320 mg every 2 week up to week 16 then every 4 weeks thereafter	16 weeks

*HS* hidradenitis suppurativa, *IL* interleukin, *NS* not specified, *SC* subcutaneous, *TNF-α* tumour necrosis factor alpha

The experts provided their opinions on the use of biological therapies to manage HS, specifically with regard to treatment initiation and the use of biosimilars (Table 3).

**Table 3:** Expert opinion on the use of biological treatments in the management of hidradenitis suppurativa

**Initiation of treatment**
Data indicate that a delay of ≥ 10 years to initiation of biological treatment is a significant risk factor for poor response [85], highlighting the importance of both early diagnosis and initiation of potent anti-inflammatory therapy early in the course of the disease
During this early ‘window of opportunity’, lesions are reversible and medical therapy is at its most effective [86]
**Use of biosimilars**
Biosimilars of adalimumab are available and may be considered as initial therapy
However, in well-controlled patients, the switch from the adalimumab originator to a biosimilar might create problems with respect to effectiveness and compliance [87, 88]
Therefore, changing therapy for patients in remission on biological maintenance treatment should be avoided, if possible
Where local pharmacoeconomic considerations mandate switching to a biosimilar, physicians should monitor patients for efficacy and adherence

### Surgical methods

Surgery is an accepted strategy in HS management. The type of surgery performed, and the required margins, are based on the severity of the disease. However, because HS is a challenging and debilitating disease, a combination of both pharmacological and surgical treatment may be necessary to achieve the best outcomes [41, 51].

Palliative procedures like incision and drainage can provide temporary relief from acute pain. However, this approach only addresses the symptoms and is associated with an almost 100% recurrence rate. Deroofing is generally preferred over incision and drainage for small lesions and is associated with lower recurrence rates (20–30%) [52].

Excision of affected tissue can be either local or wide. Local excisions of the active lesion help to control disease activity but often lead to a high recurrence rate of HS. A wide radical excision is recommended by all guidelines as a surgical intervention to treat advanced regional disease and consists of the excision of large areas of involved skin with wide margins [53, 54]. Despite recommendations of early implementation of surgical management, late diagnosis of HS often makes this an impossibility [55], and surgery is often restricted to the most advanced stages of the disease [19]. The use of surgical procedures depends on several factors, such as the extent, severity, recurrence and ease of operability of the disease, and the availability of surgical expertise [54]. Nonetheless, surgical approaches may be followed by disease relapse and may require multiple interventions [19, 53]. This aspect is especially important and indicates that surgery should be implemented early in HS to avoid widespread and severe disease, which can be challenging to manage surgically [56].

#### Combining surgery and antibiotic therapy

There are a number of benefits of combining a surgical intervention and antibiotic therapy in the management of HS to aid the control of symptoms and prevent complications. A retrospective cohort study showed that patients with HS have a high rate of postoperative infections [57], indicating that perioperative antibiotics should be administered to patients.

#### Combining surgery and biological therapy

A recent study evaluated the efficacy and safety of surgery in combination with different pharmacological treatments for HS and reported that immunomodulatory treatment can be continued both in the pre- and postoperative period without giving rise to an increased risk of complications [51].

Combining biological therapy with surgery has been shown to yield a higher effect than either biological therapy or surgical intervention alone [57, 58]. An important notion is that biological agents have been shown to reduce the inflammatory load in HS lesions [59]; the use of adalimumab and infliximab have both proven to be safe and efficacious when used peri-surgically [54].

Delphi consensus guidelines on the surgical treatment of HS recommend the use of maintenance biological therapy postoperatively, irrespective of the involvement of other anatomical sites. However, discontinuation of biological therapy can be considered in patients who achieve persistent resolution of HS after radical surgery [41].

The experts’ shared their knowledge and opinions on the role of surgery to manage HS (Table 4), where they focused on surgical techniques, use of concurrent biological therapies, timing of surgery and the role of antibiotics.

**Table 4:** Expert opinion on the use of surgery to manage of hidradenitis suppurativa

**Surgical techniques**
Surgery is a recognised strategy in HS management and can be performed on both localised lesions and advanced regional disease. Several surgical methods are available. Minor palliative interventions, such as incision and drainage or deroofing, may show around 100% and 27% of recurrence, respectively. Wide local excision may show a recurrence in around 13% of the cases. The type of surgery and margins should be determined based on the body region, the type of the lesion, and the severity of the disease [89]
Although there are no accepted definitions for surgical techniques and recurrence in HS, it is generally considered that more extensive resections are associated with a lower risk of recurrence
**Surgery in patients receive biological therapies**
If surgical intervention (major or minor) is needed in a patient on biological therapy, the biological agent should be continued during and after surgery [41]
Evidence from case series and observational studies indicates that the immunomodulatory properties of biological agents may enhance the outcomes of radical surgical resection without increasing the risk of postoperative infections [42, 43]
**Timing of surgery**
Unfortunately, many patients are referred for surgery when all other medical treatments have been tried
Rather than being a last resort, surgery should be considered early in the treatment of HS, in combination with other therapeutic options, in order to avoid extensive, demolitive surgical procedures, prolonged postoperative care and undesirable outcomes, such as anaesthesia or paraesthesia at the surgical site, wound infection, pain, scarring (including hypertrophic scars), necrosis of flaps and grafts, wound dehiscence, restricted range of motion due to scar contracture, haematoma or failure of the skin graft [90, 91]
**Role of antibiotics**
Use of targeted antibiotic therapy may have an important role as prophylaxis in the setting of major HS surgery; unfortunately, lack of evidence makes it difficult to provide recommendations
To limit the risk of antibiotic resistance, a well-coordinated, multidisciplinary approach is essential to carefully plan antibiotic administration in the pre-, peri- and postoperative periods
Dermatologists, infectious disease specialists and surgeons must work together to ensure optimal timing of antibiotic administration for the best outcomes
In the period preceding surgery, antibiotics should be used to reduce inflammation and treat or prevent bacterial infections, as needed
Perioperative antibiotic prophylaxis should be routinely administered according to local guidelines to prevent infections at the surgical site
After surgery, antibiotics should not be routinely administered unless there is evidence of infection or specific clinical indications
Randomised controlled trials and observational studies focusing on the combination of antibiotic treatment and major surgery are needed to develop novel treatment strategies

*HS* hidradenitis suppurativa

## Patient stratification and predictive biomarkers

Biomarkers, if evaluated critically, are useful tools in disease diagnosis and identification of drug targets, among others. In HS, biomarkers have the potential to improve both disease understanding and management, allowing personalised therapeutic approaches. Currently, there is a wide range of identified HS biomarkers, the expression of which has been associated with HS, but not all of them have been critically evaluated [60]. Nonetheless, the following biomarkers have emerged as important, and further validation might prove their clinical utility: (i) susceptibility/risk biomarkers: fasting serum insulin, smoking and family history; (ii) diagnostic biomarkers: serum IL-2R, IL-17, IL-1B, IL-6, IL-8 and IFN-γ, among others; (iii) monitoring biomarkers: serum IL-17, sonographic dermal vascularity, serum erythrocyte sedimentation rate and body mass index, among others; and (iv) predictive biomarkers: presence of epithelialised tunnels and a positive family history.

Grand and colleagues demonstrated that high frequency ultrasound (HFUS) could be a valid imaging-based biomarker in HS [61]. They found a strong correlation between histology and ultrasound measurements of skin layers and tunnels in patients affected by HS [61]. It has been demonstrated that the clinical examination can underestimate the severity of the disease, the HFUS leads an objective assessment allowing timely intervention and treatment [62]. The power Doppler signal, moreover, can be a valid tool for measuring the degree of inflammation in cutaneous lesions. HFUS represents the opportunity to assess the anatomical and functional changes in HS objectively and to monitor therapeutic outcomes [63].

In a critical evaluation of HS biomarkers, only four achieved a high Grading of Recommendations, Assessment, Development, and Evaluation (GRADE) rating, namely elevated serum IL-2R (diagnostic), dermal Doppler vascularity (monitoring), the presence of epithelialised tunnels and a positive family history (predictive) [60]. The authors concluded that none of the identified biomarkers has sufficient clinical validity to be recommended for routine use in the clinical setting, further highlighting the importance and need for research to enhance the value of biomarkers in the management of HS [60].

Expert opinions on the potential and current role of biomarkers and factors that contribute to HS severity in the management of HS were discussed (Table 5).

**Table 5:** Expert opinion on the role of biomarkers in the management of hidradenitis suppurativa

**Potential role of biomarkers**
The use of predictive biomarkers in HS is envisioned to have significant positive outcomes, enhancing disease comprehension and management, patient stratification and individually tailored therapies
**Smoking history and obesity**
Evidence indicates that smoking has a negative effect on the course of HS, as it has been connected with disease severity [28]
Indeed, analysis of lesional skin specimens from patients with HS showed a stronger expression of IL-17R in obese patients and those who smoked [92], and patients with HS who were non-smokers respond better to antibiotics [93]
**Disease site and other comorbidities**
Furthermore, prevalent site (i.e. genital, perianal, perineal site) and comorbidity (i.e. obesity, chronic inflammatory bowel diseases, cognitive disabilities) can further aggravate the clinical picture; as such, assessment of disease severity could be highly aided by the use of diagnostic instruments, such as ultrasound for the identification of tunnels that are not evident clinically
**Other factors**
Other factors that contribute to disease severity included disease duration, family history, syndromic/non-syndromic HS, IHS4 and IHS4-55
At present, important predictive biomarkers for the identification of patients with HS who are at risk of rapid disease progression are IL-2R, increased dermal vascularisation (at ultrasound), and ultrasound examination for the prediction of disease evolution (fibrotic and cicatricial) and therapeutic response based on the level of inflammation
Predictors of transition to severe disease include active smoking, obesity and active disease in two or three areas [94]
**Current role of biomarkers**
Despite recent advances, current biomarkers for disease progression do not have sufficient sensitivity to guide truly personalised treatment for HS and further work is required [95]

*HS* hidradenitis suppurativa, *IHS4* International Hidradenitis Suppurativa Severity Score System, *IHS4-55* International Hidradenitis Suppurativa Severity Score System, dichotomous version; *IL* interleukin, *R* receptor

## IL-17 and HS

The IL-17 family of cytokines comprises six members (IL-17A–17F) [64]. IL-17, which is mainly produced by neutrophils, mast cells and Th17 cells [51], is a potent pro-inflammatory cytokine and its involvement in immune responses to various infections, including fungal, bacterial, viral and parasitic infections [64]. Both protective and pathological functions have been attributed to IL-17 in health (i.e. wound healing, epithelial proliferation and inflammation to combat infection) and disease (i.e. chronic inflammation, pathogenic tissue remodelling and tumorigenesis), respectively [65]. However, an excessive production of IL-17 has been observed in most autoimmune conditions [66].

Th17 differentiation from uncommitted naïve T lymphocytes is driven by the presence of transforming growth factor-β and IL-6 in the local milieu; this activates the Th17-specific transcription factor retinoic acid-related orphan receptor-γ (RORγT), which is stabilised by the cytokine IL-23 [67]. Notably, these cytokines have been found to be significantly increased in the papillary and reticular derma, as well as plasma [68].

Elevations of several pro-inflammatory cytokines, including TNF-α, IL-1β, IL-23 and IL-17, have been found in HS lesions, both at the level of gene expression and mRNA expression [1, 69]. Elevated levels of serum IL-17 have been noted in patients with HS [70]. Moreover, a substantial infiltration of the immune cells highly enriched polyfunctional Th17 cells has been observed in HS lesional skin [71]. Of note, IL-17 overexpression has been found in lesional, perilesional and unaffected skin, suggesting subclinical inflammation occurring prior to the development of active lesions [22].

IL-17 plays several key roles in fuelling the pro-inflammatory process, namely through induction of monocyte and neutrophil chemotaxis in the skin, and recruitment of Th17 and myeloid cells in HS lesions throughout disease progression [11]. IL-17 also balances the actions of IL-22 on keratinocyte differentiation and epithelial cell migration [72, 73]. In fact, novel findings and identification of molecular biomarkers predicting drug responses have contributed to our understanding and highlighted a therapeutic potential for anti-IL-17 agents in HS [11].

### Mechanisms of action and therapeutic potential of interleukin-17 inhibitors

IL-17 is an important therapeutic target in several autoimmune inflammatory diseases and cancers [64]. In addition, several lines of evidence have pinpointed IL-17 as a key player in the pathogenesis of HS, paving the way for a number of targeted therapies against IL-17 in the clinical setting, including human immunoglobulin (Ig) G1/κ monoclonal antibodies (secukinumab and CJM112), a humanised IgG1 antibody (bimekizumab), a human IgG2 monoclonal antibody (brodalumab), ligand trap using Affibody^®^ molecules (Izokibep, a novel subcutaneous inhibitor with a small molecular size), and a novel trivalent nanobody (sonelokimab, neutralises IL-17A and IL17F) [11].

Of note, by targeting the IL-17 receptor A (IL-17RA) through selective binding to homodimers or heterodimers, drugs such as bimekizumab and secukinumab block downstream signalling and inhibit the inflammatory pathway; they have proven efficacy in treating HS, further expanding the frontiers in HS management and providing a rationale for targeting IL-17 in HS [11]

Overall concepts and details on the use of IL-17–targeted therapies (particularly secukinumab) for HS are presented based on the experts’ clinical experience (Table 6).

**Table 6:** Expert opinion on the role of interleukin-17–targeted therapies in the management of hidradenitis suppurativa

**Overall concepts**
In HS, an alteration of the differentiation process occurs, favouring a dysfunctional differentiation toward Th17 cells and resulting in the production of IL-17
In fact, there is increased infiltration of Th17 cells in HS skin lesions compared with non-lesional skin, which is associated with an increased level of cytokines that guide the differentiation of Th17 cells
Along with Th17 expression in lesional skin, the expression of IL-17 is also augmented, constituting the inflammatory driver of the pathogenic process
Given that the hyperproduction of IL-17 plays a crucial role throughout the course of the disease, early intervention in the pro-inflammatory phase of HS could represent the target for IL-17 inhibitors (before the formation of fibrosis) or after surgical excision of the fibrotic component (in combination with surgery), and inhibitory therapy could be directed toward several steps, including differentiation, production and receptor binding
**Role of secukinumab**
Based on current knowledge, secukinumab should be recommended as a first-line agent, equal to adalimumab, in patients with moderate-to-severe HS, including but not limited to treatment-naïve patients and those with a recent disease onset, thus exploiting the window of opportunity to change the course of disease progression [86]
Other specific patients who could benefit from secukinumab therapy are:
i) those with primary or secondary non-response to adalimumab therapy
ii) treatment-naïve patients with severe HS and elevated IHS4 at the baseline
iii) patients with ≥ 20 tunnels, although this was an exclusion criterion in the clinical trial setting
iv) patients in whom treatment with another anti-IL-17 therapy (e.g. bimekizumab) has failed; these patients can be offered an interclass switch to secukinumab therapy
v) patients treated with JAK inhibitors, either as off-label treatment for HS or for the treatment of concomitant conditions, but who do not respond to the therapy
vi) patients undergoing surgery as part of a combined treatment approach
vii) patients in whom adalimumab is contraindicated (e.g. those with heart failure)
Patients who fail treatment with adalimumab and those with more severe disease may display latency in response to secukinumab therapy [96]; therefore, caution should be exercised in interpreting the response after 16 weeks of secukinumab because there are patients who respond slowly over a longer period [97]
Patients with ≥ 20 tunnels may especially benefit from the combination of surgery and secukinumab because the anti-inflammatory effects of secukinumab can enhance the long-term efficacy of surgery
Loss of response to adalimumab occurs at about 3–6 months and onwards, so patients should receive adalimumab for ≥ 6 months before switching to secukinumab
For patients with very severe HS and a suboptimal response to adalimumab, the off-label combination of secukinumab with a TNF-α inhibitor could be considered

*HS* hidradenitis suppurativa, *IHS4* International Hidradenitis Suppurativa Severity Score System, *IL* interleukin, *JAK* Janus kinase, *Th* T helper, *TNF* tumour necrosis factor

## Role of secukinumab in HS therapy

Secukinumab, which was originally developed in 2001 by Novartis, specifically targets IL-17A and prevents it from interacting with the receptor [74]. Secukinumab is indicated for the treatment of moderate-to-severe plaque psoriasis, psoriatic arthritis, axial spondyloarthritis, enthesitis-related arthritis and juvenile psoriatic arthritis [48]. Secukinumab has also been approved for the treatment of moderate-to-severe HS in adults based on two major phase III randomised, controlled clinical trials (SUNSHINE and SUNRISE). These trials showed clinical effectiveness by rapid improvement of signs and symptoms in patients with HS administered secukinumab every 2 weeks, along with a favourable safety profile and sustained response up to 52 weeks [45].

A recently published study analysed the effect of secukinumab on patients with HS enrolled in the SUNSHINE and SUNRISE trials, comparing biological-naïve patients with those who had previously received other biological therapy [37]. Secukinumab improved the signs and symptoms of HS in both groups, indicating that it can be used as a first- or later-line biological treatment.

Additionally, a prospective single-centre study evaluated the effectiveness and safety of secukinumab in a real-life setting in 21 patients with HS [75]. HiSCR was achieved at week 16 and improved up to week 52, highlighting secukinumab as a potential treatment for patients with severe HS who had failed previous adalimumab treatment [75].

Another study, involving 67 patients, showed that secukinumab offers both safety and effectiveness in real-world clinical settings for patients with HS who are refractory to conventional systemic therapy [76]. The study compared IHS4-55 and HiSCR performance and observed an inverse correlation between disease burden and response to treatment with secukinumab at week 24.

The experts suggested that secukinumab should be recommended as a first-line agent in the treatment of HS (Table 6).

## Delayed diagnosis in HS and the significance of a MDT

Several studies have assessed the diagnostic delay in adults with HS and have shown that it ranges from 3 to 10 years after appearance of the first symptoms [36]. The rarity of HS, its diverse phenotypes and association with comorbidities (i.e. metabolic disease and diabetes mellitus, among others) contribute to delayed HS diagnoses, as do flawed understanding and lack of awareness among medical providers and patients [7, 36].

The effects of delayed diagnosis are multifaceted and can cause disease progression (with associated worsening of symptoms, pain and suffering) and a delay in the initiation of appropriate therapy in a timely manner to avoid further damage. In HS, diagnostic delay is associated with more severe disease [77], the development of local sequelae, systemic comorbidities (cardiovascular and psychiatric), reduced response to medical treatment [78] and an increased number of surgically treated sites [77].

The experts’ provided their opinions on approaches to reducing diagnostic delay in HS, including specialist care and improving patient awareness (Table 7).

**Table 7:** Expert opinion on approaches to improving the diagnosis of hidradenitis suppurativa

**Background**
Given the complexity, association with comorbidities and current lack of awareness of and education about HS, an optimal management approach requires the joint expertise of a comprehensive and MDT to ultimately reduce disease burden and improve quality of life for patients with HS
**General approaches**
Updated information, including the evolving treatment landscape, needs to be disseminated to all healthcare providers who may encounter patients with HS
Insertion of HS in the Diagnostic-Therapeutic Care Pathway of immune-mediated diseases is important
Although dermatologists might be well equipped in terms of disease recognition, access to a dermatology unit may be complicated or delayed (e.g. due to waiting lists) if the patient seeks help from another type of specialist before being referred to a dermatologist; one way to fill this gap is to provide formal education about HS to non-dermatology specialties, including internal medicine, family medicine, emergency medicine, surgery, plastic surgery, obstetrics and gynaecology, infectious disease, urology, gastroenterology and paediatrics
Importantly, general practitioners, who are often unaware of HS pathology, could help to shorten diagnostic delays through continuing medical education courses, lectures and workshops
**Specialist care**
For patients who are referred for specialist care, HS units should consist of a core of two or more dermatologists (at least a senior dermatologist and a co-worker), a plastic surgeon and a paramedic, with input from other specialists (e.g. radiologists, infectious disease specialists, gynaecologists) and healthcare professionals (e.g. psychologists, wound care nurses, nutritionists) as needed
In addition, a surgical MDT is necessary for major surgery and should be composed of radiologist (i.e. MRI expert), dermatologist and/or the plastic surgeon, proctologist, urologist or gynaecologist
Front-line providers (i.e. those who may first encounter patients with HS) include general practitioners, paediatricians and health assistants operating at regional care centres, but any of the aforementioned specialists/consultants may act as a front-line provider and refer patients to the HS unit
**Raising patient awareness**
For the general population, efforts should be aimed at raising HS awareness (e.g. through increased emphasis on social media platforms), especially to reach young audiences
The formation of HS patient support groups and foundations may be useful, since many patients are also unaware of the disease, for a better understanding of HS pathophysiology and pathogenesis, and how the disease can be recognised, as well as to facilitate access to medical care
Leaflets, posters, websites or booklets briefly explaining HS disease risk factors in a simplified manner could greatly raise awareness among the general population

*HS* hidradenitis suppurativa, *MDT*, multidisciplinary team, *MRI* magnetic resonance imaging

## Conclusions

Concerted efforts must be made to ensure a timely diagnosis and optimal treatment for patients with HS, while keeping in mind the window of treatment opportunity. Early and targeted biological therapy is necessary for effective management of inflammation and to halt disease progression, and can definitely impact the natural course of the disease. Ideally, the patient journey should include the combined efforts of a MDT, particularly when determining the optimal therapeutic approach and considering the combination of medical and surgical therapies, since early combination treatment is needed to achieve the best therapeutic outcomes. As such, it is important that patients with HS are referred to and treated at hospitals or centres of excellence equipped with providers with different specialties, such as surgeons, in order to offer patients multidisciplinary management. The clinical phenotype of HS must guide the therapeutic choice and the combination of both medical and surgical therapies. Finally, education of both medical professionals and the general population can help to facilitate early diagnosis and treatment of HS.

## Data Availability

No datasets were generated or analysed during the current study.
